# Histone deacetylase inhibitor SAHA mediates mast cell death and epigenetic silencing of constitutively active D816V KIT in systemic mastocytosis

**DOI:** 10.18632/oncotarget.14181

**Published:** 2016-12-25

**Authors:** Katarina Lyberg, Hani Abdulkadir Ali, Jennine Grootens, Matilda Kjellander, Malin Tirfing, Michel Arock, Hans Hägglund, Gunnar Nilsson, Johanna Ungerstedt

**Affiliations:** ^1^ Immunology and Allergy Unit, Department of Medicine Solna, Karolinska Institutet and clinical immunology and transfusion medicine, Karolinska University Hospital, Stockholm, Sweden; ^2^ Department of Medicine Huddinge, Karolinska Institutet and Hematology Center, Karolinska University Hospital, Stockholm, Sweden; ^3^ Mastocytosis Center Karolinska, Karolinska University Hospital and Karolinska Institutet, Stockholm, Sweden; ^4^ Molecular and Cellular Oncology, LBPA CNRS UMR 8113, Ecole Normale Supérieure de Cachan, Cachan, France and Laboratoire Central d’Hématologie, Groupe Hospitalier Pitié-Salpêtrière, Paris, France; ^5^ Department of Medical Sciences, Uppsala University and Section of Hematology, Uppsala University Hospital, Uppsala, Sweden

**Keywords:** systemic mastocytosis, epigenetics, KIT, chromatin, mast cells

## Abstract

Systemic mastocytosis (SM) is a clonal bone marrow disorder, where therapeutical options are limited. Over 90% of the patients carry the D816V point mutation in the KIT receptor that renders this receptor constitutively active. We assessed the sensitivity of primary mast cells (MC) and mast cell lines HMC1.2 (D816V mutated), ROSA (KIT WT) and ROSA (KIT D816V) cells to histone deacetylase inhibitor (HDACi) treatment. We found that of four HDACi, suberoyl anilide hydroxamic acid (SAHA) was the most effective in killing mutated MC. SAHA downregulated KIT, followed by major MC apoptosis. Primary SM patient MC cultured *ex vivo* were even more sensitive to SAHA than HMC1.2 cells, whereas primary MC from healthy subjects were less affected. There was a correlation between cell death and SM disease severity, where cell death was more pronounced in the case of aggressive SM, with almost 100% cell death among MC from the mast cell leukemia patient. Additionally, ROSA (KIT D816V) was more affected by HDACi than ROSA (KIT WT) cells. Using ChIP qPCR, we found that the level of active chromatin mark H3K18ac/H3 decreased significantly in the KIT region. This epigenetic silencing was seen only in the KIT region and not in control genes upstream and downstream of KIT, indicating that the downregulation of KIT is exerted by specific epigenetic silencing. In conclusion, KIT D816V mutation sensitized MC to HDACi mediated killing, and SAHA may be of value as specific treatment for SM, although the specific mechanism of action requires further investigation.

## INTRODUCTION

Systemic mastocytosis (SM) is a rare clonal bone marrow disease, previously defined as a subtype of myeloproliferative neoplasms but has recently been redefined as an own disease entity in the 2016 WHO classification of hematological malignancies [1]. SM is characterized by elevated numbers of mast cells (MC) in one or more tissues, almost always including the bone marrow, and enhanced levels of MC mediators, typically tryptase and histamine [2, 3]. Symptoms originate from the MC burden itself and/or MC released mediators. SM may be indolent (ISM), with no impact on life expectancy, or aggressive (ASM) with a survival of 1-2 years. Mast cell leukemia (MCL), which is the most aggressive category of SM, is characterized by > 20% of malignant circulating MCs and has the worst prognosis of all SM categories, median overall survival being less than 6 months. Management options are limited to symptomatic treatment, and there is no curative therapy [2, 4, 5], except for allogeneic stem cell transplantation, however the majority of ASM patients are elderly and not eligible for stem cell transplant [6].

The development, growth and survival of MC are dependent on the binding of stem cell factor (SCF) to the tyrosine kinase receptor KIT [7, 8]. More than 90% of SM patients carry a point mutation (most commonly D816V) in the kinase domain of the KIT receptor rendering the receptor constitutively active [9]. D816V mutated SM is resistant to treatment with tyrosine kinase inhibitors like Imatinib [10], however two recent studies with the novel broad tyrosine kinase inhibitor Midostaurin shows promising data with an overall response rate of 60% in patients with aggressive forms of SM [11, 12]. KIT receptor mutations are considered essential for the development of SM, however little is known about the regulation of *KIT* expression in normal or neoplastic human MC.

Epigenetic changes include DNA methylation and posttranslational modifications of histones, the regulation of which is frequently perturbed in myeloid malignancies such as the myelodysplastic syndromes and myeloproliferative neoplasms [13]. In recent years, several point mutations in genes encoding epigenetic regulators have been detected in such malignancies, with implications for their pathogenesis, progression and prognosis [14, 15]. Similar mutation patterns are also present in SM [16, 17] and accumulating evidence indicates that improved understanding of these recurrent mutations allow prediction of development towards aggressive disease phenotypes [18–21].

Histone deacetylase inhibitors (HDACi) are small molecules of various structure background of which many are in clinical trials for solid and hematological tumors, and a few are already approved for some specific tumor subtypes [22]. The HDACi suberoyl anilide hydroxamic acid (SAHA) is a pan-inhibitor of class I and II HDACs, and in addition also affects acetylation of non-histone proteins, of which one of the most extensively studied is HSP90 [23, 24]. Treatment with SAHA alters the expression of 5-15% of protein coding genes, depending on cell type [22, 25, 26]. SAHA induces apoptosis in malignant cells [27] and is currently in phase I-III clinical trials for treatment of a variety of solid and hematological malignancies, and is approved for treatment of cutaneous T cell lymphoma, as is Romidepsin in the US. Recently, Panobinostat, another HDACi similar in function to SAHA, was approved as third line treatment in a combination regimen, for myeloma. In human GIST tumors that frequently carry KIT mutations, however not commonly the D816V mutation, SAHA and other HDACi have been shown to decrease KIT mRNA levels and acetylate HSP90, abrogating its activity as a KIT chaperone [24]. Additionally, the HDACi AR-42 has been described to downregulate constitutively active KIT in malignant murine and canine mast cells [28], although the underlying mechanism remains unclear.

The purpose of this study was to characterize the effect of HDACi in human SM cells. We demonstrate that in human SM cell lines carrying the D816V mutation, SAHA downregulates KIT mRNA followed by decreased KIT protein levels, cell surface KIT expression and apoptosis, and that the mechanism is at least partially via epigenetic silencing. In addition, we show that primary mast cells from SM patients are highly sensitive to SAHA-induced cell death, whereas normal bone marrow mast cells are resistant. Thus, HDACi may be a potential clinical treatment option for SM patients.

## RESULTS

### HDACi reduces HMC1.2 growth and viability

**Figure 1:**
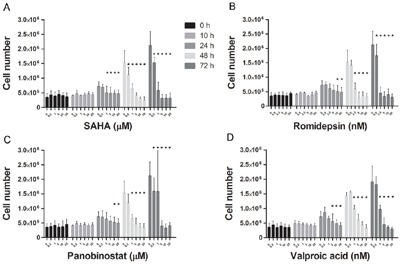
HDACi induces HMC1.2 mast cell line growth inhibition There was a dose- and time-dependent inhibition of cell growth upon culture of HMC1.2 cell line with: **A**. SAHA, **B**. Romidepsin, **C**. Panobinostat and **D**. Valproic acid. Triplicates of three biological replicates were analyzed. The earliest and also most profound effects were seen upon treatment with SAHA. * indicates significance on the level of p<0.05.

**Figure 2:**
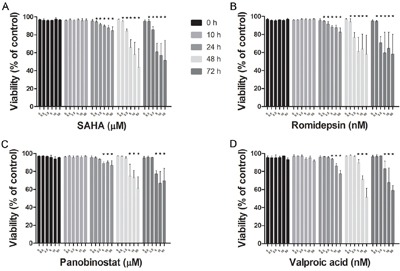
Decrease in HMC1.2 mast cell line viability after treatment with HDACi There was a dose- and time-dependent decrease in cell viability of HMC1.2 cells by: **A**. SAHA, **B**. Romidepsin, **C**. Panobinostat and **D**. Valproic acid. Viability was determined by Annexin V and PI staining, and triplicates of three biological replicates were analyzed. Of the four HDACi, SAHA was the most efficient in decreasing cell viability. * indicates significance on the level of p<0.05.

SAHA, Romidepsin and Panobinostat, three inhibitors of class I and II HDACs, and Valproic acid, an inhibitor of class I HDACs, were assessed for dose- and time-dependent effects on HMC1.2 growth and viability. All HDACi induced a dose- and time-dependent decrease in HMC1.2 growth (Figure 1A-1D), with a concomitant decrease in cell viability (Figure 2A-2D). As SAHA induced an earlier and more profound decrease in both growth and viability, we used SAHA for further investigations in our study.

### SAHA reduces KIT expression and induces apoptosis in HMC1.2 cells

**Figure 3:**
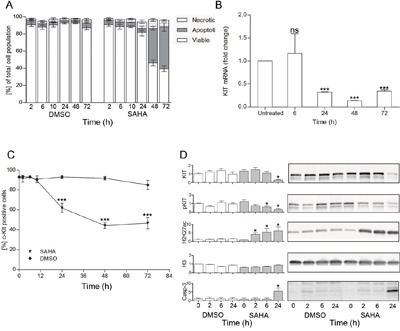
SAHA induces mast cell apoptosis, down-regulation of KIT and increase in H3K27ac **A**. Effects of SAHA on cell death revealed a significant increase of apoptotic cell death at 48 h (p<0.01), as determined by flow cytometry by Annexin V and propidium iodide staining, n=3; **B**. A significant decrease of KIT mRNA was seen at 24 h (p<0.05, duplicates of 4 individual experiments), as was **C**. a decrease in the percentage of cell surface KIT positive cells (p<0.01 at 24 h,48 h and 72 h compared to baseline as well as to DMSO treated cells (triplicate of two separate experiments); **D**. Western blot analyses showed early responses to SAHA with increased H3K27ac already at 2 h, and decrease in phosphorylated KIT at 6 h, whereas total KIT decreased and active caspase 3 increased significantly at 24 h. Total histone H3 remained unchanged by SAHA treatment. *=p<0.05, **=p<0.01.

Viability was further analyzed by flow cytometry in Annexin V and propidium iodide stained cells. We found that the decrease in viability was mainly due to a profound increase in apoptotic cell death at 48 h (p<0.01, Figure 3A). Already at 24 h there was a significant decrease of KIT mRNA (p<0.01, Figure 3B), as well as a significant reduction in the percentage of high surface KIT positive cells (p<0.01 at 24 h, Figure 3C). Thus, the decrease in growth, KIT mRNA and cell surface KIT clearly preceded the apoptotic cell death. When assessing protein levels, we observed a significant increase in active histone mark H3K27ac already at 2 h and a decrease in phosphorylated active KIT at 6 h (p<0.05, Figure 3D), indicating an early onset of SAHA mediated effects. Total KIT protein level was significantly decreased at 24 h, co-occurring with the decrease in percentage KIT positive cells, and KIT mRNA. Cleaved caspase 3 was also significantly increased at 24 h as a first sign of apoptosis induction (p<0.05, Figure 3D).

### Additional targets of SAHA in HMC1.2 cells

**Figure 4:**
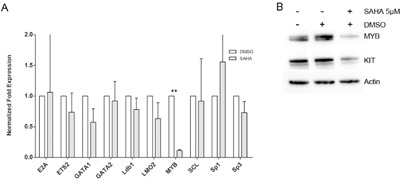
SAHA effects on transcription factors Ten transcription factors potentially regulating human KIT were assessed for transcript levels after SAHA treatment. SAHA treatment significantly decreased MYB mRNA levels **A**. and on protein level, where a representative blot is depicted **B**. White bars are DMSO treated control, grey bars indicate SAHA treatment. **=p<0.01.

We thereafter set out to investigate transcription factors potentially regulating KIT expression. We chose ten transcription factors known as KIT regulators in mouse models, or proposed as regulators of human KIT, namely E2A, ETS2, GATA1, GATA2, Ldb1, LMO2, MYB, SCL, Sp1 and Sp3, and assessed the transcript levels of these transcription factors after 24 h culture of HMC1.2 with 5 μM SAHA. We found that SAHA induced a significant decrease in MYB. (Figure 4A). The downregulation of MYB by SAHA was also confirmed at protein level by western blot, where a representative blot is shown in Figure 4B. Thus, SAHA downregulates transcription factor MYB at mRNA and protein level, and MYB may be one contributing factor in the downregulation of KIT exerted by SAHA.

### SAHA reduces active phosphorylated KIT and mediates apoptosis in ROSA ^KIT WT^ but even more profound in ROSA ^KIT D816V^ cells

**Figure 5:**
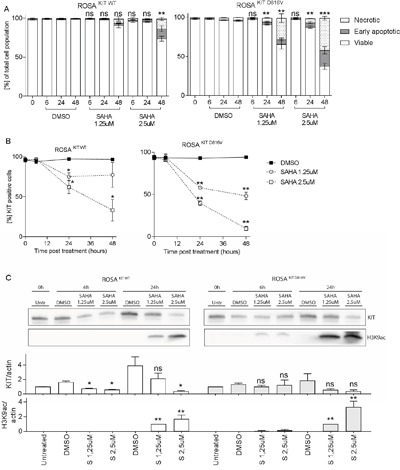
SAHA effects on ROSA ^KIT WT^ and ROSA ^KIT D816V^ cells **A**. SAHA induced a dose- and time-dependent decrease in viability, mainly due to apoptotic cell death, in ROSA cells. The effects were seen earlier and at lower SAHA concentrations in ROSA ^KIT D816V^, where a significant decrease in viability was seen already at 24 h with 1.25 μM SAHA, compared to ROSA ^KIT WT^. **B**. There was a significant decrease in percentage KIT positive cells in both cell lines in response to SAHA, and also here ROSA ^KIT D816V^ were more profoundly affected by SAHA. **C**. A significant increase in H3K9ac was seen for both ROSA ^KIT WT^ and ROSA ^KIT D816V^ cells. KIT was significantly decreased in ROSA ^KIT WT^ cells, however note that in the DMSO control, KIT is increasing significantly at 6 and 24 h compared to baseline, thus the results are difficult to interpret. For ROSA ^KIT D816V^, there is a tendency to decrease in KIT at 24 h for both SAHA doses. *=p<0.05, **=p<0.01, *** = <0.001.

ROSA ^KIT WT^ and ROSA ^KIT D816V^ were cultured with 1.25 and 2.5μM SAHA for up to 48h. A significant decrease in viability was seen already at 24h for 1.25 μM SAHA for ROSA ^KIT D816V^ cells, whereas ROSA ^KIT WT^ cells needed 2.5 μM SAHA for 48h in order for SAHA to achieve a significant decrease in cell number (Figure 5A). As for HMC1.2 cells, the decrease in viability was mainly due to apoptotic cell death, and apoptosis occurred earlier and more profoundly in ROSA ^KIT D816V^ compared to ROSA ^KIT WT^ cells (Figure 5A), indicating that SAHA may have a selective effect on D816V mutated cells. Similarly, percentage surface KIT positive cells decreased more profoundly in ROSA ^KIT D816V^ cells compared to ROSA ^KIT WT^ (Figure 5B).

When assessing protein levels, we observed a slight, however not significant, increase in active histone mark H3K9ac already at 6h in ROSA ^KIT D816V^ and a significant increase in H3K9ac in ROSA ^KIT WT^ as well as ROSA ^KIT D816V^ at 24h also in the lower SAHA concentration (Figure 5C). We also measured total KIT protein however total KIT protein had a strong propensity to increase in the DMSO sample already at 6h and profoundly at 24h, likely due to dense culturing of cells, and there was a significant variation between the 3 biological replicates, which influences the statistical analysis. However, there was a significant decrease in total KIT in ROSA ^KIT WT^ (where KIT increased in the DMSO control) but not in ROSA ^KIT D816V^ treated cells (where KIT did not increase in the DMSO control) at 6h and 24h with the higher SAHA concentration (p<0.05).

### Primary SM patient mast cells are sensitive to SAHA treatment

**Figure 6:**
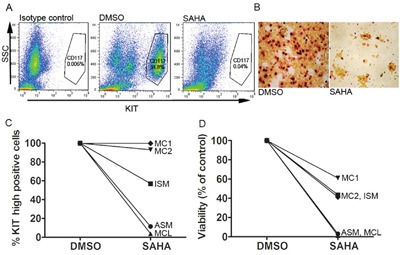
SAHA inhibits KIT-expression and induces cell death in primary SM patient mast cells **A**. Flow cytometry gating strategy for side scatter (SSC) and KIT expression (CD117), example of gating for CD117 positive cells. **B**. Staining of isolated bone marrow MC from a patient with MCL reveals a profound loss of tryptase positive cells upon incubation with 5μM SAHA 48h. **C**. Response of primary SM patient MC and healthy volunteer MC cultured *ex vivo* with 5 μM SAHA for 48 h, show a decrease in surface KIT in all patients, with a more profound decrease the more severe form of SM: ASM and MCL. Non-mutated healthy age matched bone marrow mast cell KIT expression was unaffected by SAHA treatment. **D**. MC viability shows more cell death induced by SAHA in the more severe forms of SM: ASM and MCL. In the severe SM cases, almost all cells were dead at 48 h of SAHA treatment. Healthy age-matched bone marrow mast cell viability was less affected by SAHA.

To investigate the effect of SAHA on primary MC from three SM patients, one patient with ISM, one patient with ASM, and peripheral blood MC from one patient with mast cell leukemia (MCL), as well as bone marrow MC from two age-matched healthy controls, were treated with SAHA at 5 μM for 48 hours. Patient characteristics are described in Table 1. It is noteworthy that the patient with MCL did not have the classical D816V mutation, but a D820Y KIT mutation. Magnetic bead separated CD117 positive mast cells (confirmed as MCs by tryptase staining) were treated for 48 h with 5 μM SAHA or DMSO control and analyzed by flow cytometry gating on forward scatter and CD117 high positivity. An example of gating strategy is shown in Figure 6A. Staining of bone marrow SM MCs revealed a distinct decrease in the number of tryptase positive cells upon SAHA treatment (Figure 6B).

**Table 1:** Characteristics of the SM patients

	Patient 1	Patient 2	Patient 3
Diagnosis	SM-AHNMD	ISM	MCL
Gender	Male	Male	Female
Hemoglobin (mg/dL)	11.6	14.5	8.3
Leukocytes (x109/L)	4.4	8.4	13.5
Platelet count (x109/L)	188	229	24
*Major criteria* (mast cell aggregates)	+	+	+
*Minor criteria:* s-tryptase (μg/L) (cutoff < 20 μg/L)	180*	22	640
*Minor criteria:* BM MC CD2/25 pos	+	+	Not done
*Minor criteria:* KIT mutation	D816V	D816V	D820Y
*Minor criteria:* Aberrant mast cell phenotype	-	+	-

*serum tryptase is not applicable when SM patient has an associated AHNMD. + =present, - absent.

SAHA induced a dramatic reduction of MCs expressing high surface KIT, especially in the case of more aggressive disease phenotypes, without affecting surface KIT in the healthy control MCs (Figure 6C). Cell death was close to 100% in the ASM and MCL patients, less profound in the ISM patient and the healthy control MCs (Figure 6D).

### SAHA induces specific epigenetic alterations in the KIT gene of HMC1.2 cells

**Figure 7:**
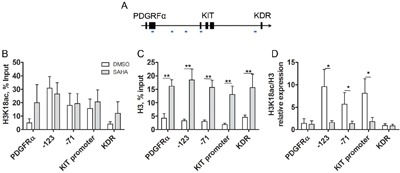
SAHA induces histone acetylation in mast cells **A**. Schematic diagram of ChIP qPCR primers over the KIT gene, as well as control genes upstream and downstream of KIT, the PDGFRα and KDR genes, respectively. **B**. ChIP qPCR of active histone mark H3K18ac (n=4) shows high levels in active KIT, and low levels in control genes. Control gene active H3K18ac increases upon SAHA treatment however the increase was not statistically significant. **C**. In response to SAHA, histone H3 density increased significantly in the KIT gene as well as in the upstream and downstream genes, PDGFRα and KDR (p<0.01 for all regions, n=5). **D**. ChIP qPCR for H3K18ac corrected for H3 density, H3K18ac/H3 revealed that H3K18ac/H3 was high at baseline indicating active chromatin, and decreased significantly in all regions of the KIT gene upon SAHA treatment (p<0.05 for -71, -123 and KIT promoter region), implying specific epigenetic regulation of the KIT gene. The active marks were low in PDGFRα and KDR. White bars are DMSO treated control, grey bars indicate SAHA treatment.

To assess whether the SAHA-induced apoptosis involves epigenetic regulation of the KIT gene, we next treated HMC1.2 cells with 5 μM SAHA for 24 h, the early 24 h time point chosen so that major apoptosis would be avoided. At 24 h, samples were subjected to ChIP with H3K18ac active chromatin mark. Following ChIP, qPCR was performed with primers covering the KIT promoter and two upstream sites in the KIT gene (chosen based on modifications according to Ensemble (http://www.ensembl.org) and UCSC (https://genome.ucsc.edu), as well as two control genes, PDGFRα upstream of KIT, and KDR downstream of KIT (probes schematically drawn in Figure 7A).

In untreated HMC1.2 cells, the level of H3K18ac was high in all regions of the KIT gene, indicating active chromatin, as expected. This was not further increased by SAHA treatment (Figure 7B), likely due to saturation of H3K18ac. In contrast, in adjacent genes PDGFRα and KDR, H3K18ac was low in untreated cells indicating inactive genes, and upon SAHA treatment the genes were acetylated, thus activated, following the predicted pattern of SAHA in increasing histone acetylation. The same pattern in PDGFRα and KDR was seen when the experiment was repeated in healthy unmutated bone marrow MC (n=2), however for these healthy MC, we observed as expected that H3K18ac was low at baseline in all regions of KIT, indicating inactive KIT, and increased upon SAHA treatment (results not shown).

Unexpectedly, we observed a significant increase in total histone H3 density upon SAHA treatment, not only in the KIT region but also in PDGFRα and KDR (Figure 7C). This was however not a general genome wide phenomenon, as the total levels of histone H3 assessed by western blot, remained unchanged upon SAHA treatment (Figure 3D). For accuracy, we re-calculated the levels of H3K18ac relative to the H3 density. This method has been employed by others finding varying total histone H3 [29]. The levels of H3K18ac/H3 were high at baseline, indicating active chromatin, and decreased significantly in all regions of the KIT gene upon SAHA treatment (p<0.05 for each region in the KIT gene -123, KIT promoter and -71, Figure 7D), indicating that SAHA induces a selective silencing of the KIT gene.

To confirm the findings of specific epigenetic effects on KIT compared to surrounding genes, we repeated the treatment of HMC-1.2 with 5 μM SAHA for 24 h, thereafter ChIP was performed with antibodies against active (H3K27ac) and repressive (H3K27me3, H3K9me3) chromatin marks, as well as histone H3 for histone density (n=2-4). For active chromatin mark H3K27ac, we found a similar pattern to active chromatin H3K18ac, with high H3K27ac levels in the KIT region of untreated cells, which increased significantly in response to SAHA treatment, both in KIT, PDGFRα and KDR (Supplementary Figure 1A). Accordingly, the levels of the repressive marks H3K9me3 and H3K27me3 were low in the KIT region of untreated HMC1.2 cells, indicating an active gene, and remained unchanged in response to SAHA (Supplementary Figure 1B, 1C). These changes are all in accordance with what is expected regarding the pattern of histone modifications in active genes, and with the general effects of SAHA treatment. In untreated HMC1.2 cells, the repressive chromatin marks were high in PDGFRα and KDR, and the level of active chromatin marks were low, all parameters indicating that PDGFRα and KDR are silent in untreated cells (Supplementary Figure 1). Upon SAHA treatment, the level of active chromatin mark H3K27ac increased in PDGFRα as well as KDR indicating activation, and the repressive marks remained largely unchanged (Supplementary Figure 1). This is in accordance with SAHA exerting its effects as an inhibitor of histone deacetylases, thus increasing histone acetylation. When assessing the levels of active chromatin H3K27ac/H3, there was a significant decrease of active chromatin mark in the KIT promoter region, but not in the control genes, thus showing the same pattern as H3K18ac/H3, supporting the finding of an epigenetic shut down of KIT. In the repressive histone marks relative to H3 density, H3K9me3/H3 and H3K27me3/H3 were low at baseline and remained low in the KIT region, indicating active chromatin throughout (Supplementary Figure 2B, 2C). In contrast, the repressive chromatin marks in the control genes PDGFRα and KDR were high at baseline and decreased upon SAHA treatment in H3K27me3 (Supplementary Figure 2B, 2C) thus showing a similar pattern as the repressive marks non-adjusted for H3 density, further implying a specific epigenetic silencing of the KIT gene by SAHA. Taken together, these findings indicate that the constitutively active D816V-mutated KIT characteristic for SM is downregulated by SAHA through epigenetic mechanisms involving reduction in the levels of active chromatin marks H3K18ac/H3 and H3K27ac/H3 specifically in the KIT promoter region. This subsequently leads to MC apoptosis in KIT-mutated cell line HMC1.2 and in SM patient cells, whereas healthy MCs are much less affected.

## DISCUSSION

In the current study, we demonstrate that several HDACi namely SAHA, Panobinostat, Romidepsin and Valproic acid, all induce a dose- and time-dependent growth arrest and cell death of KIT-mutated HMC1.2 cells. The response was most profound for SAHA. Further, SAHA treatment rapidly increased total acetylated histones already after 2 hours of treatment, as well as a decrease in active phosphorylated KIT at 6 hours. This quick increase of acetylated histones is in line with findings in the literature [30]. At 24 h, there was a decrease in KIT mRNA levels, total KIT protein as well as cell surface KIT, followed later by major mast cell apoptosis. The direct causal relationship between KIT mRNA and protein decrease to apoptosis induction remains to be studied, however the events occurred in a distinct time line making a causal relation plausible, and may imply that a decrease in active KIT may have a causative role in the SAHA mediated cell death. Additionally, when comparing ROSA ^KIT WT^ and ROSA ^KIT D816V^ cells, the SAHA-mediated effects were consistently more profound in ROSA ^KIT D816V^ cells.

Furthermore, we show for the first time that primary SM patient KIT positive MCs decrease in cell surface KIT and undergo major cell death in response to SAHA, whereas healthy age-matched bone marrow MC are less affected. Interestingly, cells from the patients with more aggressive SM disease were more sensitive to *ex vivo* SAHA treatment than cells from ISM and healthy MC and in fact, cells from a patient with highly aggressive MCL were all dead after 48 hours of SAHA exposure. Clinically, these findings are of considerable interest, since there are very limited treatment options for patients with aggressive SM [2, 4–6]. Notably, the ISM and ASM patients both carried the D816V KIT mutation, whereas in the MCL patient we found a more uncommon KIT D820Y mutation. This mutation is also in the intracellular, 2^nd^ catalytic domain close to D816V, and is reported in around 1% of GIST [31] and melanoma [32] tumors. As D816V, D820Y is associated with imatinib resistance [31, 32]. Thus, the SAHA mediated killing seems specific to malignant mast cells when compared to healthy, more profound in KIT mutated compared to WT cells, seemingly regardless of which specific KIT mutation is present.

In addition, we examined the potential effect of SAHA on histone modifications in the KIT gene and control genes PDGFRα upstream and KDR downstream of KIT, by ChIP-qPCR with antibodies to active and repressive chromatin marks. Upon SAHA treatment, histone acetylation marks increased as expected. Surprisingly, we found that upon SAHA treatment of HMC1.2, histone H3 density increased significantly in the KIT region as well as in adjacent genes PDGFRα and KDR. This was not seen in the chromatin in general, as the total histone H3 content remained unchanged. To correct for the histone H3 density, we recalculated the histone H3 chromatin marks relatively to the total H3 content in each region of each sample, as has been previously performed in studies with variation in H3 content [29].

In all three regions of the KIT gene, SAHA treatment significantly decreased the active chromatin mark H3K18ac/H3 content. This was also seen for H3K27ac in the promoter region of KIT, and is important as H3K27ac specifically marks active promoters [33]. This response was unique to the KIT region, and was not seen in the PDGFRα and KDR control genes, where H3K18ac/H3 and H3K27ac/H3 levels remained unchanged after SAHA treatment. The decrease in active chromatin mark indicates a specific epigenetic response to SAHA closing the chromatin in the KIT gene, coherent with our current finding of SAHA downregulating KIT mRNA and protein levels. Although this epigenetic regulation and specifically the mechanism behind the increase in H3 density requires further studies, *e.g*., by whole genome sequencing at different early time points of treatment and ChIP sequencing or ATAC sequencing with H3, we hypothesize that the regulation may be exerted by SAHA-induced epigenetic activation of an hitherto unknown upstream negative regulator of the KIT gene.

Altogether, our findings show that SAHA induces apoptotic cell death in mast cell lines as well as in SM patient MCs, whereas healthy bone marrow MCs are less sensitive. SAHA treatment downregulates active phosphorylated KIT, followed by decreased KIT mRNA, protein and surface KIT expression. We demonstrate a decrease in active histone marks in the KIT region in response to SAHA treatment, indicating that SAHA exerts a specific epigenetic downregulation of KIT. Although the epigenetic mechanism of action of SAHA as well as the mutated mast cell selectivity herein described requires further investigations, we have demonstrated that mast cells from aggressive SM exhibit high sensitivity to SAHA suggesting that SAHA may be of clinical use for treatment of SM.

## MATERIALS AND METHODS

The study was approved by the local Ethics Committee of Stockholm. Samples from indolent and aggressive SM patients were obtained in connection with routine diagnostic bone marrow sampling, while in the case of the MCL patient, a peripheral blood sample was obtained in conjunction with routine blood sampling, all with oral consent. After oral consent was obtained from healthy (no hematological disorder) individuals scheduled to undergo hip replacement surgery, some of the bone marrow that is removed routinely during surgery was collected for our study. These healthy control samples were confirmed by PCR to be D816V negative.

### Culture of mast cell line HMC1.2

The human mast cell line HMC1.2 [34] was cultured at 37°C in a humidified atmosphere with 6% CO2, in Iscove’s modified Dulbecco’s medium (IMDM) supplemented with 10% fetal bovine serum (FBS, Thermo Fischer Scientific, Waltham, MA, USA), 2 mM L-glutamine (Sigma Aldrich, St Louis, MO, USA), 100 μg/mL streptomycin (Sigma Aldrich), 100 IU/mL penicillin (Sigma Aldrich), and 1.2 mM 1-thioglycerol (Sigma Aldrich). Experiments were only performed when cells were in logarithmic growth phase and were over 95% cell surface KIT positive.

### Culture of WT and D816V mutated cord blood derived ROSA mast cells

The human mast cell lines ROSA ^KIT WT^ and ROSA ^KIT D816V^ [35] were cultured in IMDM supplemented with 10% fetal calf serum, 2 mM L-glutamine, 100 μg/mL streptomycin, 100 IU/mL penicillin. ROSA KIT WT cells where grown with additional 80 ng/mL murine stem cell factor.

### Primary culture of mast cells from SM patients and healthy donors

Bone marrow samples were first incubated in ammonium chloride buffer for erythrocyte lysis, and thereafter sorted using Miltenyi CD117 microbead kit according to the manufacturer’s protocol. For culture experiments, the cells were maintained in StemPro-34 SFM medium supplemented with 100 ng/ml rhSCF (SOBI, Stockholm, Sweden) and 10 ng/ml rhIL-6 (PeproTech EC Ltd, Rocky Hill, USA). Enzymatic tryptase staining was applied to assess the purity of the mast cells, and if less than 50% were tryptase positive, a second round of magnetic bead CD117 separation was performed. All cells were cultured at 37°C in a humidified atmosphere with 6% CO2. The viability of patient and healthy mast cells was evaluated with trypan blue exclusion using the Countess® automated cell counter (Invitrogen).

### HDACi treatment, cell viability and KIT expression

Cell line and primary patient or healthy mast cells were treated with SAHA (Cayman Chemical, Ann Arbor, Michigan), Panobinostat (Sigma Aldrich) or Romidepsin (Sigma Aldrich), all dissolved in DMSO, or Valproic acid (Sigma Aldrich) dissolved in distilled water. Control experiments were incubated with DMSO vehicle alone. Viability determination was done with either trypan blue exclusion or using flow cytometry Annexin V-FITC propidium iodide Apoptosis detection Kit according to the manufacturer’s protocol (eBioscience). For quantification of KIT expression the HMC-1.2 cells were incubated with 1% human CD117 (A36E2) (Bioledgend) while for the patient samples we used CD117 (A3C6E2)-PE (Miltenyi Biotec, Bergisch Gladbach, Germany) or 1% mouse IgG2b isotype control PE (Bioscience, San Jose, CA, USA) for 15 min at 4°C. Enriched CD117 high positive bone marrow cells have previously been described to be highly enriched for mast cells, thus we defined CD117 high positive cells as mast cells [36]. All samples were analyzed on a FACSCalibur flow cytometer (Becton Dickinson), employing the Cellquest software, and the data acquired analyzed with the FlowJo 7.6 program.

For ROSA cells, apoptosis staining was performed with Annexin V and 7-AAD (BD Bioscience) in Annexin V Binding Buffer (BD Bioscience).

### Western blot

Cells were lysed with RIPA buffer on ice, centrifuged and supernatant protein content quantified. Proteins were separated on SDS-polyacrylamide gel electrophoresis (Mini-PROTEAN TGX Precast Gels, Bio-Rad, Hercules, USA); followed by transfer onto a nitrocellulose membrane (Trans-Blot Turbo Mini Nitrocellulose Transfer Packs). The membrane was blocked in Odyssey Blocking buffer (LI-COR, Lincoln, Nebraska, USA) prior to overnight incubation at 4°C with one of the following primary antibodies: anti-KIT (D12A3), Caspase 3, phospho-KIT and anti-H3K27Ac (Cell Signaling, Danvers, USA), anti H3 and H3K9ac (Abcam, Cambridge, UK). Subsequently, for HMC1.2 cells, membranes were incubated simultaneously with secondary fluorescent anti-rabbit and anti-mouse antibodies labeled with spectrally distinct IRDye^®^ fluorescent dyes (wave lengths 680 nm and 800 nm; LI-COR), and bands were quantified in the Licor development apparatus with Image Studio 4.0 software (LI-COR). Western blots where analysed and quantified using Image Studio Software version 2.1.10 (LICOR). The protein of interest was normalized to the loading control, β-actin (DM1A, Sigma) or α-tubulin (AC-15, Cell signaling). For ROSA cells, membranes were incubated with HRP-conjugated IgG secondary antibody (Cell Signaling, Danvers, USA), proteins visualized with chemiluminescence (GE Healthcare) in a ChemiDoc MP System (Bio-Rad) using Image Lab 4.1. Bands were normalized to β-actin and vehicle control.

### RNA extraction and qPCR

Total RNA was extracted using Trizol (ThermoFischer Scientific) according to the manufacturer’s instructions, with the addition of a second precipitation with absolute ethanol. cDNA was synthesized using Maxima Reverse transcriptase (ThermoFisher Scientific) according to the producers protocol. The subsequent qPCR was performed using 200 nM primer (TaqCopenhagen, Copenhagen, Denmark) and SybGreen (ThermoFisher Scientific) on the StepOnePlus Real-Time PCR system (Applied Biosystems). Normalized gene expression was calculated using the CFX manager software (Bio-Rad). The expression was normalized to housekeeping gene GAPDH, and to the DMSO vehicle control.

### Transcription factor analysis

Transcription factors to analyze were chosen by extensive literature search. HMC-1.2 cells were treated with 5 μM SAHA or vehicle control for 24 hours, whereafter total RNA was extracted with Trizol and cDNA was synthesized using iScript (Bio-Rad) according to the manufacturer’s protocol. Real time PCR was performed using 100 ng cDNA, 200 nM primer (TaqCopenhage) and SybGreen (Bio-Rad) on the CFX96 Touch™ Real-Time PCR. Gene expression normalized to housekeeping gene was calculated using the CFX manager software (Bio-Rad), and thereafter normalized to the DMSO vehicle control. Primer list is shown in Supplementary Table 1.

### Chromatin immunoprecipitation followed by qPCR (ChIP-qPCR)

ChIP assay was performed using the iDeal Chip-seq kit from Diagenode (Denville, USA) according to the manufacturers procedure. Briefly, after 24 h incubation with or without SAHA, 10^6^cells were crosslinked, whereafter crosslinking was quenched and nuclei isolated. Chromatin was fragmented using the Bioruptor sonicator (Diagenode) for 25 min (30 second pulses) to produce fragments of 200-500 nucleotides. Antibodies used for ChIP were: H3K27Ac (Cell Signaling), H3K27Me3 (Cell Signaling), H3K9Me3 and H3K18Ac (Abcam). Immunoprecipitates were collected using protein A-coated magnetic beads from the iDeal Chip-seq kit (Diagenode). The precipitated DNA was eluted on a rotating wheel for 30 minutes, and crosslinking was reversed by overnight incubation at 65°C, whereafter DNA was extracted with a PCR purification kit (MiniElute, Qiagen, Hilden, Germany), and qPCR using the primers specific for target gene loci (*KIT*) was performed. Primers were for KIT promoter forward 5′GCCTTTTCCGTGATCCATTCA3′, reverse 5′GGCGACGAGATTAGGCTGTTA3′, -71 kb of KIT forward 5′ TGCCAGAACATCATTGATATCTCTG’3; reverse 5′CAGGCATGTGGTTGTTAGATATTGT’3; -123 kb forward 5′ ATG GCT TCT GTC CTT GGA GC’3; reverse 5′ GGC CTG ACT GCT TAC TCC TC‘3, PDGFRα forward 5′ CGCGGTTTTTGAGCCCATTA’3, reverse 5′ATGGAGTAAAGAGCGTGCCC’3, KDR forward 5′ GCCTTCAGATGCCACAGACT’3, and reverse 5′ TGGCAGCCATATGGAGTTGG’3. For qPCR, 200 nM primer (TaqCopenhage) and SybGreen (Bio-Rad) on the CFX96 Touch™ Real-Time PCR Detection System (Bio-Rad) was used. Normalized gene expression was calculated using the CFX manager software (Bio-Rad). Enrichment was validated by qPCR with primers specific for the KIT gene including sequences in its promoter, as well as a gene upstream (PDGFRα) and downstream (KDR) of KIT. The expression was normalized to the percentage of input sample, that is a part of the sample not subjected to ChIP.

### Statistical analyses

Student’s paired two-sided t-test was applied to compare treated and untreated samples, with * indicating statistical significance on the level of p<0.05, and ** on the level of p<0.01. For multiple comparisons between treated and untreated samples over time, ANOVA repeated measures comparing means was applied.

## SUPPLEMENTARY MATERIALS FIGURES AND TABLES


