# Supplementary material for: Histone deacetylase inhibitor SAHA mediates mast cell death and epigenetic silencing of constitutively active D816V KIT in systemic mastocytosis
Source: Oncotarget. 2016 Dec 25;8(6):9647–59. doi: 10.18632/oncotarget.14181 (PMC5354760; doi:10.18632/oncotarget.14181)
Supplement: Supplementary file 1 [file oncotarget-08-9647-s001.pdf]

Histone deacetylase inhibitor SAHA mediates mast cell death and epigenetic silencing of constitutively active D816V KIT in systemic mastocytosis

SUPPLEMENTARY FIGURES AND TABLE

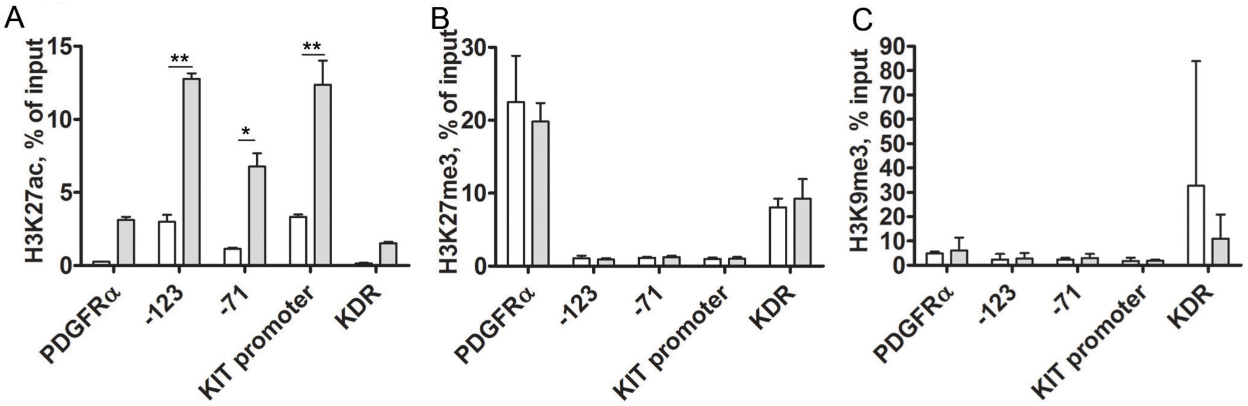

Supplementary Figure 1: ChIP qPCR for active H3K27ac and repressive H3K27me3 andH3K9me3 histone marks. **A.** ChIP qPCR of active histone mark H3K27ac shows high levels of active histone mark in KIT and low levels in control genes, all, increasing upon SAHA treatment (n=2,); **B.** ChIP qPCR of repressive chromatin H3K27me3 shows high levels in repressed control genes and low levels in the KIT gene, with no change upon SAHA treatment (n=2); and **C.** ChIP qPCR of repressive chromatin H3K9me3 shows the same pattern as repressive H3K27me3, repressed control genes with high H3K9me3 and low H3K9me3 in active KIT, with no change upon SAHA treatment (n=2). White bars are DMSO treated control, grey bars indicate SAHA treatment.

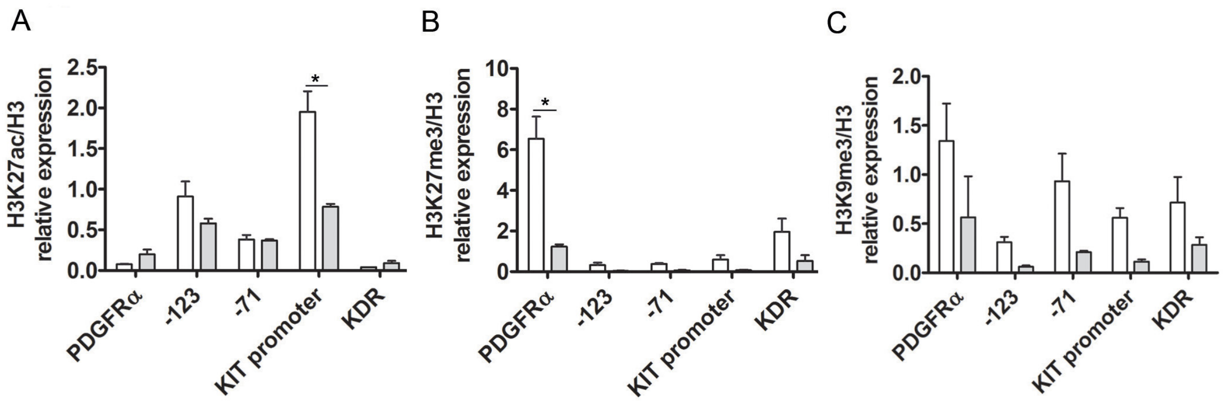

Supplementary Figure 2: ChIP qPCR of histone marks corrected for H3 density. A. Active histone mark H2K27ac/H3 shows an increase in active marks in the control regions, although not significant however only tested twice (n=2). As for H3K18ac/H3, KIT promoter region decreases significantly upon SAHA treatment indicating a specific shut down of KIT upon SAHA treatment ($p<0.05$, n=2). **B.** Repressive H3K27me3/H3 decreases significantly in repressive marks in PDGFRα, indicating activation of chromatin upon SAHA treatment (n=2, $p<0.05$), and **C.** repressive H3K9me3/H3 shows very low levels of repressive histone marks (n=2). White bars are DMSO treated control, grey bars indicate SAHA treatment.

Supplementary Table 1: Primers for RT PCR of potential transcription factors regulating KIT

Gene	Forward 5´to 3´	Reverse 3´to 5´
GAPDH	GAAGGTGAAGGTCGGAGTCAAC	CAGAGTTAAAAGCAGCCCTGGT
ETS2	AGTCTGGTGAACGTGAATCTG	CGGAGGTGAGGTGTGAATTTT
c-MYB	GAGGTGGCATAACCACTTGAA	AGGCAGTAGCTTTGCGATTTC
Ldb-1	CCGTTACTTTAGCACTGTGTTTG	CCGTGATGGATGAGTTGTGGTA
GATA1	CTGTCCCCAATAGTGCTTATGG	GAATAGGCTGCTGAATTGAGGG
GATA2	ACTGACGGAGAGCATGAAGAT	CCGGCACATAGGAGGGGTA
LMO2	AAGCGGATTCGTGCCTATGAG	AGTTGATGAGGAGGTATCTGTCA
E2A	ACAGCTCTCTGAAATGGGGC	AGGAGTGAAGGACAGGGTGT
SLC	GAAGACCATCCGACTTGCTTATC	GGATCAAAGCGATCTACTGTCAA
Sp1	GGAGTTGGTGGCAATAATGG	CTCCTCCTCCAGTGCTGCT
Sp3	AGTGGGCAGTATGTTCTTCCC	GACTGGATCTGTGGTATCACTTG
